# A framework for analyzing DNA methylation data from Illumina Infinium HumanMethylation450 BeadChip

**DOI:** 10.1186/s12859-018-2096-3

**Published:** 2018-04-11

**Authors:** Zhenxing Wang, XiaoLiang Wu, Yadong Wang

**Affiliations:** School of Computer Science and Technology, Harbin Institute of Technology, Harbin, 150001 China

**Keywords:** DNA methylation, Illumina 450K, Normalization, Ontology interpretation

## Abstract

**Background:**

DNA methylation has been identified to be widely associated to complex diseases. Among biological platforms to profile DNA methylation in human, the Illumina Infinium HumanMethylation450 BeadChip (450K) has been accepted as one of the most efficient technologies. However, challenges exist in analysis of DNA methylation data generated by this technology due to widespread biases.

**Results:**

Here we proposed a generalized framework for evaluating data analysis methods for Illumina 450K array. This framework considers the following steps towards a successful analysis: importing data, quality control, within-array normalization, correcting type bias, detecting differentially methylated probes or regions and biological interpretation.

**Conclusions:**

We evaluated five methods using three real datasets, and proposed outperform methods for the Illumina 450K array data analysis. *Minfi* and *methylumi* are optimal choice when analyzing small dataset. BMIQ and RCP are proper to correcting type bias and the normalized result of them can be used to discover DMPs. R package *missMethyl* is suitable for GO term enrichment analysis and biological interpretation.

## Background

DNA methylation is an important epigenetic modification which has shown numerous associations with biological processes and complex diseases such as diabetes, schizophrenia and cancer [1–4]. However, the methylomic landscape in disease pathogenesis has not yet been well characterized, especially in cancer where DNA methylation can be altered dramatically. Interests of exploring the associations between DNA methylation and complex diseases increase in disease studies.

Illumina Infinium HumanMethylation450 (450K) BeadChip array, which covers over 480K CpG sites and targets 96% of CpG islands in human genome [5], has been widely utilized in many large studies, such as The Cancer Genome Atlas (TCGA) and The International Cancer Genome Consortium (ICGC) Project [6]. With the availability of public data resources, a number of methods for analyzing the Illumina 450K array data became rapidly available in the past few years.

Unlike the previous platform Illumina Infinium HumanMethylation27 (27K) BeadChip, in which only one probe type is utilized, the Illumina 450K BeadChip includes two distinct probe types, Infinium I (n=135501) and Infinium II (n=350076) [5]. Each CpG site of Infinium I is targeted by two 50bp probes: one for detecting “methylated” (M) intensity and one for detecting “unmethylated” (U) intensity, whereas each CpG site of Infinium II uses just one probe to distinguish “M” and “U” intensity through different dye colors (green and red), then the *β*-value, indicating the methylation level of one CpG site, can be computed as *β*=*M*/(*M*+*U*+*α*) where *α* is 100 generally. M-value, *M*=*log*_2_(*β*/(1−*β*)), the logit-transformed *β*-value, is another quantity used in following up analysis.

**Fig. 1:**
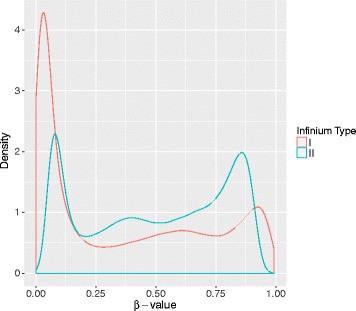
Density curves of the *β*-values of Infinium I and Infinium II of a HPV-HNC sample (GSM937820 in GSE38268). The distribution models of *β*-values of the two types’ probes are different, where the Infinium II probe’s curve shows a narrower dynamic range

Owing to the platform design, more loci could be tested simultaneously on a fixed array size. Bibikova et al. [5] report a difference between Infinium I and Infinium II that Infinium II assays demonstrate an average of *β*-value upward shift for U and downward shift for M (shown in Fig. 1). Dedeurwaerder et al. [7] further evaluated the Illumina 450K BeadChip and reported that the *β*-values obtained from Infinium II probes had a narrower dynamic range and were less reproducible than those obtained from Infinium I leading to a type design bias. Hence, data preprocessing and normalization is critical for analyzing the Illumina 450K array data. Although many methods and R packages for correcting probe design bias have been proposed [8–14], more attention should be paid on the entire framework for analyzing Illumina 450K array data and selecting different modules on various datasets. Here we present a generalized framework including importing data, quality control, within-array normalization, correction of the type bias, identification of differential methylated probes or regions and biological interpretation, whilst the most popular modules for each step will be introduced, facilitating users to select appropriate module according their needs.

## Methods

**Fig. 2:**
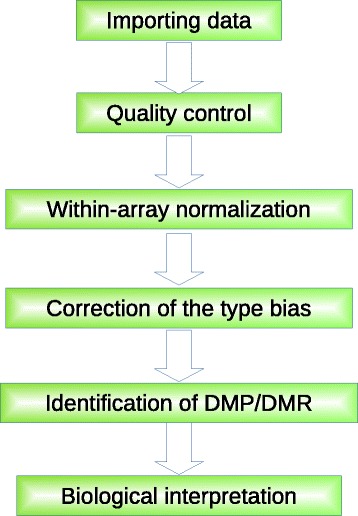
A generalized framework of Illumina 450K array data analysis. There are six steps in the generalized framework leading to a successful methylation data analysis

Figure 2 shows the diagram of the framework and the detail of each step will be introduced below.

### Importing data

There are mainly two forms of the Illumina 450K array data: i) raw (*.idat) data which is the direct output from Illumina iScan system and stores intensities for each probe, ii) *.txt data, which is usually got after simple preprocessing and is easier to access. Both file formats can be handled by R packages: *.idat files can be read by *illuminaio* package [15] and *.txt files can be dealt with *minfi* [12], *wateRmelon* [11] et al. We use three datasets for expounding our framework.

*Dataset 1:* Illumina 450K dataset from Dedeurwaerder et al. (GEO accession number: GSE29290) [7]. Three samples on HCT116WT cell-lines are considered, to evaluate the capability of methods to reduce the replicate variation.

*Dataset 2:* Illumina 450K dataset of fresh frozen head and neck cancer (HNC) samples form GSE38268 [16], where three of them are HPV+ and other three are HPV- samples.

*Dataset 3:* Illumina 450K dataset of level 1 methylation data of TCGA KIRC samples, which are *.idat files containing 160 normal samples and 325 tumor samples, to evaluate the efficiency of different modules.

### Quality control

After the data imported into R, we would evaluate the quality of data. First, probes displaying a high detected *p-value* should be filtered out (e.g. > 0.01), while such probes have a *β*-value of “NA” in *.txt files. It is worth mentioned that different strategies of assigning missing *β*-value are applied in different modules. In *minfi*, the *β*-values are assigned with “NAs” when both M and U intensities are zero, but an additional criteria is that “NAs” will be assigned if either M or U intensities don’t fluoresce above background. Second, there are 850 inbuilt control probes on the 450K array, such as bisulfite conversion I, bisulfite conversion II, extension, hybridization and negative (n=613), which can be used to evaluate the other probes’ intensities. Samples that can’t pass this quality control are excluded in further analysis. Third, probes on Chromosome X or Y should be filtered out to eliminate the impact of sex on differential methylated analysis in many studies. Fourth, Price ME et al. [17] reported that there were 4.3% of Illumina 450K probes containing a known SNP at the targeted site. Such probes should cause problems in inter-sample analysis. Last but not the least, cross-reactive probes on the Illumina 450K array are identified depending on [18], which is particularly problematic because the *β*-value of such probes is more likely to represent a combination of multiple sites and not the level of initially targeted CpG sites.

### Within-array normalization

This step includes background correction and color bias adjustment. Background correction methods have been developed by Triche et al. [19] such as Noob and Normexp, which are based on convolution models and use out-of-band (OOB) probes intensities to measure the background. The *lumi* R package provides two different methods for eliminating the background. The first one is based on the negative-control probes inbuilt the BeadChip and the second one estimates the background from the density modes of probes intensities. The second one would show bad performance when there are more than two density modes for some sample. The popular *methylumi* R package [20] proposed a color bias adjustment based on smooth quantile or shift-and-scaling normalization. Globally, these methods seem to improve the data quality in some cases [7].

### Correction of the type bias

The type bias is the one that is most crucial to correct as it is the main source decreasing the data quality. There have been several efforts to develop methodologies to correct the probe type bias because of the differences between Infinium I and Infinium II. Because Infinium I probes are more stable and reproducible across different samples, most methods reduce the bias of Infinium II rather than Infinium I probes.

The first method is called peak-based correction (PBC) [7], which rescale the methylation values of Infinium II to the same modes for distribution of methylation values of Infinium I. But this method is sensitive to the shape of *β*-value density curves and is therefore less robust when the methylation density distribution does not exhibit well-defined peaks.

Touleimat and Tost [8] developed a method called Subset Quantile Normalization (SQN) based on an assumption that the *β*-values of CpGs form the same biological category should have the same density distribution. They found that the normalization result of using the “relation to CpG” annotation perfectly corrected the bias.

Subset-quantile Within Array Normalization (SWAN) [10] was developed based on the assumption that the *β*-values distribution should be the same when the probes have the same number of CpGs. But SWAN also alters Infinium I probe data, which increases Infinium I technical variation, and does not seem to improve the data quality when applied to some datasets [21].

Beta Mixture Quantile normalization (BMIQ) [9] method decomposes the density profile of Infinium I and Infinium II probes by fitting a beta-mixture model of three states: unmethylated (default *β*-value <0.25), hemimethylated (default 0.25 ≤*β*-value <0.75) and fully methylated (default *β*-value ≥0.75). Then it uses a quantile normalization to fit *β*-values distribution of Infinium II to the corresponding *β*-values distribution of Infinium I. This method does not depend on unceremonious choices of biological characteristics to be used to normalize data. Thus it seems more suitable than other methods. However, some points appear worse after BMIQ correction.

Another method, called Regression on Correlated Probes (RCP) [14], uses a quantile linear regression model of correlation between pairs of nearby Infinium I and II probes that share the same genomic context to adjust the methylation levels of Infinium II probes. The weakness of RCP is that it may not fit some experimental data leading to a result worse than raw data.

While background is important for measuring absolute methylation levels for single sample/condition experiments, we ignore the background here in this analysis since it can be cancelled out when comparing two conditions. As shown by other studies [22], widely used normalization process, which is based on the assumption that the majority of signals should not change across compared conditions, usually makes mistakes when it was applied to experiments where large portions of signals are differentially expressed. Thus, with a good quality control on the analyzed datasets, we didn’t choose to apply common normalization strategies.

It is also important to remove non-biological variation called batch effects existing between batches and samples. Such batch effects can influence on measurement of global level that could be partially removed through between-sample normalization using principal component analysis.

### Identification of DMPs/DMRs

As we mentioned above, the main focus of many methylation studies has been on detecting differentially methylated probes (DMPs) or regions (DMRs) associated with a phenotype. The *β*-value is the default value for methylation measurement, allowing easy biological interpretation. Another type of value, M-value, is used to express the degree of methylation obtained with Infinium. Due to the heteroscedasticity of *β*-value, the variance of M-value across the methylation range is approximately constant, so the M-value has better statistical properties. The two types of value are used in different methods.

SQN simply considers a probe as DMP if the absolute value of the difference between *β*-value medians of paired samples is higher than 0.2:

$$\left|median\left({\beta^{N}_{1},\ldots,\beta^{N}_{n}}\right)-median\left({\beta^{T}_{1},\ldots,\beta^{T}_{n}}\right)\right|\ge0.2 $$

where $\beta ^{N}_{i}$ and $\beta ^{T}_{i}$ corresponding *β*-values of paired normal and tumor samples. The 0.2 threshold represents approximately a difference in methylation level of 20% which can be detected by the Infinium technology with 99% confidence [5]. Then the corresponding differentially methylated gene identities can be obtained from the list of DMPs.

*Minfi* offers a comprehensive package to analyze Illumina 450K array data, where candidate regions are determined for DMR analysis and locally weighted scatterplot smoothing (LOESS) is adopted to smooth the methylation differences between groups within each determined region. It also can find long-range alterations such as identified hypomethylated blocks [23] based on “open sea” probes. A empirical Bayes moderated t-test is used in *limma* [24] when sample sizes are less than 10, in which case M-values should be used as they rely much more on Gaussianity assumption [25].

Generally, DMRs are detected by applying various statistical techniques such as Fisher’s exact test [26, 27], t-test [27], Wilcoxon rank sum test [28] or different regression models [29–31].

### Biological interpretation

There is a long list of significant CpGs to be interpreted after differential methylation analysis. Using the Infinium annotation file, Illumina 450K probes are classified according to their relations to CGIs and to the closest annotated gene. Regarding their relation to CGIs, the probes are classified into four categories: sites located inside a CGI, sites located in the CGI shores (0-2k bp), sites located in the CGI shelves (2-4k bp) and sites located in the “open sea”. As regards their relation to annotated genes, the sites are categorized as inside the promoter, inside the 5’-UTR region, inside the gene body and inside the 3’-UTR region. Then the significant DMPs can be marked with their related genes.

Performing gene set analysis is a popular way to understand the affected potential gene pathways. Although gene set analysis is well established in gene expression experimental, the research in methylation data is ongoing in different groups. In Illumina 450K array, the numbers of CpGs associated with each gene ranges largely from 1 to 1299 [32]. Genes with larger numbers of probes are more likely to have significant differentially methylated CpGs [33]. With the ontology and knowledgebase developing [34–40], researchers can easily annotate the genes containing DMPs or DMRs to ontology entries, which brings convenience for understanding the function of genes in the pathogenesis of diseases. Obviously, a phenotype is associated with several -omics data, such as mRNA expression and protein expression, which suggests researchers should utilize integrated analysis with multi-dimension data like TCGA project does [41, 42].

## Results

### Reduce the technical variation and type bias

We evaluated the different modules of correcting methods including SQN, SWAN, Dasen, BMIQ and RCP methods on Dataset 1, described in Table 1. The methylated and unmethylated intensities are imported into R environment and some results are displayed in following figures. For each method, we first plotted the density curves of *β*-value for three samples in Dataset 1. Then, we computed the standard deviation across the three replicates.

**Table 1:** Normalization methods for Illumina 450K array data

Method	Object	R Package	Ref.
SQN	MethyLumiSet	wateRmelon	[8, 11]
SWAN	RGChannelSet	minfi,wateRmelon	[11, 12]
Dasen	MethylSet	wateRmelon	[11]
BMIQ	*β*-value, MethylSet	ENmix	[9, 13]
RCP	MethylSet	ENmix	[13]

**Fig. 3:**
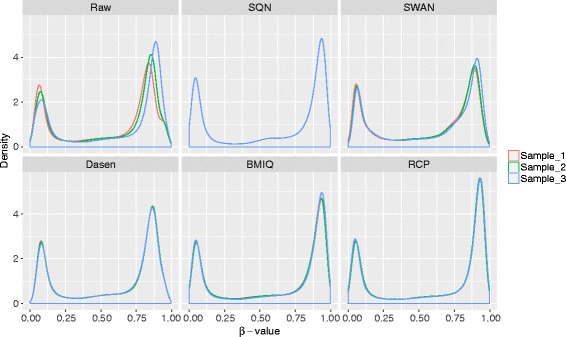
Density curves of *β*-value of replicates. Density curves of all probes after normalization using different methods are shown in this figure. The separate color represents replicates of the dataset. Raw data shows that there are differences among the replicates, while SQN, Dasen and RCP significantly reduce the technical variation

**Fig. 4:**
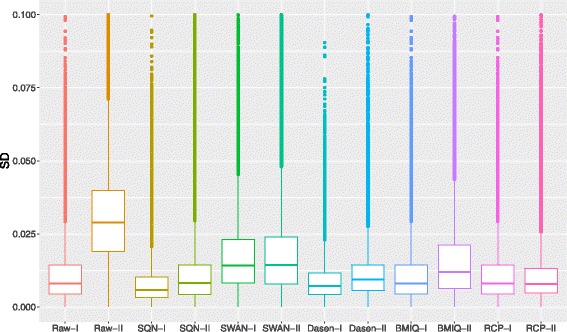
Box plot of standard deviation across replicates. The standard deviation between replicates after normalization shows that SQN, Dasen and RCP make the normalized *β*-values of replicates more similar than other two methods

As seen, SQN, Dasen and RCP can significantly reduce the technical variation (Fig. 3) and the same result can be seen base on standard deviation of replicates (Fig. 4). Because BMIQ and RCP do not change the *β*-values of Infinium I probes, the standard deviation of Infinium I probes of the two methods stays the same as the raw data. SWAN shows the least ability of reducing the variation among replicates.

**Fig. 5:**
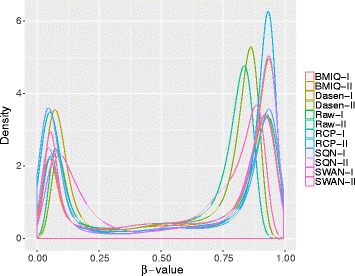
Density curves of *β*-value of two types in sample GSM815136 for different methods. Density curves of *β*-values of Infinium I/II probes are plotted with different colors. BMIQ, RCP and SQN can correct the type bias efficiently on this sample

We also plotted the density curves of *β*-value of Infinium I/II probes for different methods. BMIQ, RCP and SQN show similar performance on the sample GSM815136 such that the Infinium I and Infinium II probes have more similar distribution modes with same local maximum values, while Dasen and SWAN underperformed others regarding removing the type bias (Fig. 5).

### Identify DMPs/DMRs by preprocessed results of different methods

**Fig. 6:**
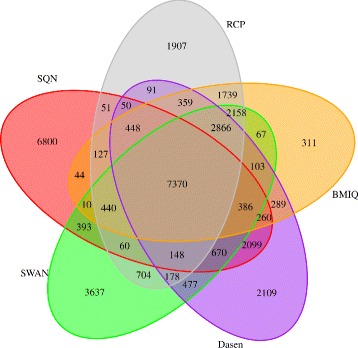
Venn diagram of DMPs obtained by five methods. After filtering probes with *p-value* <0.01, we use *dmpfinder* in *minfi* to identify DMPs with *p-value* <0.05. BMIQ and RCP can lead to better results than other three methods

**Fig. 7:**
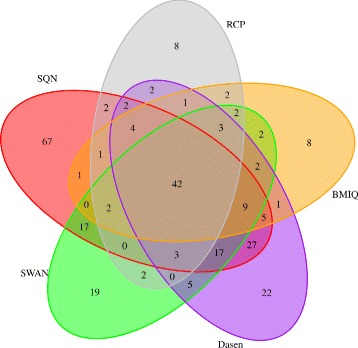
Venn diagram of GO terms obtained by five methods. There are 42 common GO terms identified by five methods. RCP has the highest rate of common terms (42/76=55.26*%*). SQN discovers most terms but 33.67% of them are identified by only this method

We used the *dmpFinder* function in *minfi* R package to evaluate the result of identifying DMPs obtained by the five methods. We selected the probes with *p-value* of the results obtained after *dmpFinder* less than 0.05 as DMPs. The number of DMPs of five methods is listed in Table 2. Then the Venn diagram of the detected probes was plotted, shown in Fig. 6. As shown in Fig. 6, BMIQ and RCP methods show larger overlap with other methods, where the ratio of common probes is 43.41% (7370/16977 in BMIQ) and 39.42% (7370/18696 in RCP). Moreover, the most probes identified after BMIQ have intersections with others and only 311 probes (311/16977=1.83*%*) are identified uniquely. But it is also shown that there are differences among results of different methods, which maybe caused by the models they applying to normalization. Then the GO term enrichment analysis were analyzed with *gometh* function in *missMethyl* package. The GO terms of FDR ≤0.05 of five methods were got (see Table 2) and the Venn diagram of GO terms obtained after normalization is shown in Fig. 7. RCP got the highest rate of common (42/76=55.26*%*) GO terms among the five methods and SQN got the most number (n=199) of GO terms of which 33.67% (67/199) identified by only this method.

**Table 2:** The number of obtained DMPs and GO terms by five methods

	SQN	SWAN	Dasen	BMIQ	RCP
*#*DMPs	19356	19667	17903	16977	18696
*#*GO term	199	125	145	85	76

### Efficiency of large dataset analysis

We use Dataset3 to evaluate the performance of *minfi* and *meffil* [43] in term of importing large data. It took 48 min and less than 3 Gb memory to import data of 485 samples (970 *.idat files) using *meffil* package on a computer with 8 Gb of RAM and 4 processors while *minfi* could not run on the same computer. *Minfi* took two hours and ∼ 23 Gb memory on a server to import the same dataset.

## Discussion

In this study we propose a generalized framework for Illumina 450K data analysis. We evaluate five methods for correcting type bias. Analysis of reducing technical replicates showed that different methods optimized different assessment criterion. The summary table of evaluation is shown in Table 3, where the score is set to 1 if there is significant change using the method, otherwise the score is set to 0.5. RCP gets the highest total score based on mentioned criteria.

**Table 3:** Summary of evaluation of five methods

	Replicate	Standard	Type bias	DMPs	GO terms	Total
	variation	deviation				
SQN	1	1	1	0.5	0.5	4
SWAN	0.5	0.5	0.5	0.5	0.5	2.5
Dasen	1	1	0.5	0.5	0.5	3.5
BMIQ	0.5	0.5	1	1	1	4
RCP	1	1	1	1	1	5

If there is significant change, score of the method is set to 1, otherwise the score is set to 0.5

The SQN, Dasen and RCP methods could significantly improve the replicated data quality, while SWAN and BMIQ didn’t show improvement of the replicates. SWAN and Dasen didn’t remove type bias as other methods, which might be due to that models they applying cannot fit the distribution of Infinium I well as other methods.

When evaluation focused on detecting DMPs, BMIQ and RCP got more overlapped DMPs and credible GO terms than other methods. It is should pointed out that result may vary largely when using different datasets, which will be validated in further work.

Illumina MethylationEPIC BeadChip [44] microarrays have been used in some project, which contain more probes on a single array. More efficient tools are in urgent need of merging 450K and EPIC array data and the efficiency of analysis should be considered. *Minfi* has been utilized widely but it cannot handle large dataset on personal computer in our view while the newly package *meffil* displayed surprising performance.

During the evaluating processing, there were conflicts between R packages, for example, the MethylSet object in wateRmelon and minfi are different because the one in *minfi* has been updated in the newest version while the *wateRmelon* still use the previous object constructor. It is should be noticed in case of using different version of them.

## Conclusions

It is suggested that the Illumina 450K users should choose proper strategy about importing data, background eliminating, correcting dye bias, correcting the type bias and detecting DMPs or DMRs. When analyzing small dataset, *minfi* and *methylumi* are optimal choice to import data and SQN, BMIQ and RCP may be proper to correcting the Infinium I/II bias. R package *missMethyl* is suitable for GO term enrichment analysis and biological interpretation. In our view, *minfi* is a proper R package to import data, eliminate background and *ENmix* package can be used to correct the type bias, then the normalized data should be used in the remaining steps of the framework.

## References

[1] Koga Y, Pelizzola M, Cheng E, Krauthammer M, Sznol M, Ariyan S, Narayan D, Molinaro AM, Halaban R, Weissman SM (2009). Genome-wide screen of promoter methylation identifies novel markers in melanoma. Genome Res.

[2] Teng M, Balch C, Liu Y, Li M, Huang TH, Wang Y, Nephew KP, Li L (2012). The influence of cis-regulatory elements on dna methylation fidelity. PloS ONE.

[3] Esteller M (2007). Cancer epigenomics: Dna methylomes and histone-modification maps. Nat Rev Genet.

[4] Irizarry RA, Ladd-Acosta C, Wen B, Wu Z, Montano C, Onyango P, Cui H, Gabo K, Rongione M, Webster M (2009). The human colon cancer methylome shows similar hypo-and hypermethylation at conserved tissue-specific cpg island shores. Nat Genet.

[5] Bibikova M, Barnes B, Tsan C, Ho V, Klotzle B, Le JM, Delano D, Zhang L, Schroth GP, Gunderson KL (2011). High density dna methylation array with single cpg site resolution. Genomics.

[6] Consortium ICG (2010). International network of cancer genome projects. Nature.

[7] Dedeurwaerder S, Defrance M, Calonne E, Denis H, Sotiriou C, Fuks F (2011). Evaluation of the infinium methylation 450k technology. Epigenomics.

[8] Touleimat N, Tost J (2012). Complete pipeline for infinium®; human methylation 450k beadchip data processing using subset quantile normalization for accurate dna methylation estimation. Epigenomics.

[9] Teschendorff AE, Marabita F, Lechner M, Bartlett T, Tegner J, Gomez-Cabrero D, Beck S (2012). A beta-mixture quantile normalization method for correcting probe design bias in illumina infinium 450 k dna methylation data. Bioinformatics.

[10] Maksimovic J, Gordon L, Oshlack A (2012). Swan: Subset-quantile within array normalization for illumina infinium humanmethylation450 beadchips. Genome Biol.

[11] Pidsley R, Wong CC, Volta M, Lunnon K, Mill J, Schalkwyk LC (2013). A data-driven approach to preprocessing illumina 450k methylation array data. BMC Genomics.

[12] Aryee MJ, Jaffe AE, Corrada-Bravo H, Ladd-Acosta C, Feinberg AP, Hansen KD, Irizarry RA (2014). Minfi: a flexible and comprehensive bioconductor package for the analysis of infinium dna methylation microarrays. Bioinformatics.

[13] Xu Z, Niu L, Li L, Taylor JA (2015). Enmix: a novel background correction method for illumina humanmethylation450 beadchip. Nucleic Acids Res.

[14] Niu L, Xu Z, Taylor JA (2016). Rcp: a novel probe design bias correction method for illumina methylation beadchip. Bioinformatics.

[15] Smith ML, Baggerly KA, Bengtsson H, Ritchie ME, Hansen KD (2013). illuminaio: An open source idat parsing tool for illumina microarrays. F1000Research.

[16] Lechner M, Fenton T, West J, Wilson G, Feber A, Henderson S, Thirlwell C, Dibra HK, Jay A, Butcher L (2013). Identification and functional validation of hpv-mediated hypermethylation in head and neck squamous cell carcinoma. Genome Med.

[17] Price EM, Cotton AM, Lam LL, Farré P., Emberly E, Brown CJ, Robinson WP, Kobor MS (2013). Additional annotation enhances potential for biologically-relevant analysis of the illumina infinium humanmethylation450 beadchip array. Epigenetics Chromatin.

[18] Chen Y-a, Lemire M, Choufani S, Butcher DT, Grafodatskaya D, Zanke BW, Gallinger S, Hudson TJ, Weksberg R (2013). Discovery of cross-reactive probes and polymorphic cpgs in the illumina infinium humanmethylation450 microarray. Epigenetics.

[19] Triche Jr TJ, Weisenberger DJ, Van Den Berg D, Laird PW, Siegmund KD (2013). Low-level processing of illumina infinium dna methylation beadarrays. Nucleic Acids Res.

[20] Davis S, Du P, Bilke S, Triche T, Bootwalla M. methylumi: Handle illumina methylation data. R Package version 2.0. 2014.

[21] Dedeurwaerder S, Defrance M, Bizet M, Calonne E, Bontempi G, Fuks F (2013). A comprehensive overview of infinium humanmethylation450 data processing. Brief Bioinforma.

[22] Robinson MD, Oshlack A (2010). A scaling normalization method for differential expression analysis of rna-seq data. Genome Biol.

[23] Berman BP, Weisenberger DJ, Aman JF, Hinoue T, Ramjan Z, Liu Y, Noushmehr H, Lange CP, van Dijk CM, Tollenaar RA (2012). Regions of focal dna hypermethylation and long-range hypomethylation in colorectal cancer coincide with nuclear lamina-associated domains. Nat Genet.

[24] Smyth GK (2005). Limma: linear models for microarray data. Bioinformatics and Computational Biology Solutions Using R and Bioconductor.

[25] Zhuang J, Widschwendter M, Teschendorff AE (2012). A comparison of feature selection and classification methods in dna methylation studies using the illumina infinium platform. BMC Bioinformatics.

[26] Stockwell PA, Chatterjee A, Rodger EJ, Morison IM (2014). Dmap: differential methylation analysis package for rrbs and wgbs data. Bioinformatics.

[27] Warden CD, Lee H, Tompkins JD, Li X, Wang C, Riggs AD, Yu H, Jove R, Yuan Y-C (2013). Cohcap: an integrative genomic pipeline for single-nucleotide resolution dna methylation analysis. Nucleic Acids Res.

[28] Wang D, Yan L, Hu Q, Sucheston LE, Higgins MJ, Ambrosone CB, Johnson CS, Smiraglia DJ, Liu S (2012). Ima: an r package for high-throughput analysis of illumina’s 450k infinium methylation data. Bioinformatics.

[29] Hansen KD, Langmead B, Irizarry RA (2012). Bsmooth: from whole genome bisulfite sequencing reads to differentially methylated regions. Genome Biol.

[30] Park Y, Figueroa ME, Rozek LS, Sartor MA (2014). Methylsig: a whole genome dna methylation analysis pipeline. Bioinformatics.

[31] Hebestreit K, Dugas M, Klein H-U (2013). Detection of significantly differentially methylated regions in targeted bisulfite sequencing data. Bioinformatics.

[32] Phipson B, Maksimovic J, Oshlack A (2015). missmethyl: an r package for analyzing data from illumina’s humanmethylation450 platform. Bioinformatics.

[33] Geeleher P, Hartnett L, Egan LJ, Golden A, Raja Ali RA, Seoighe C (2013). Gene-set analysis is severely biased when applied to genome-wide methylation data. Bioinformatics.

[34] Cheng L, Jiang Y, Wang Z, Shi H, Sun J, Yang H, Zhang S, Hu Y, Zhou M (2016). Dissim: an online system for exploring significant similar diseases and exhibiting potential therapeutic drugs. Sci Rep.

[35] Cheng L, Sun J, Xu W, Dong L, Hu Y, Zhou M (2016). Oahg: an integrated resource for annotating human genes with multi-level ontologies. Sci Rep.

[36] Peng J, Lu J, Shang X, Chen J (2017). Identifying consistent disease subnetworks using dnet. Methods.

[37] Cheng L, Yang H, Zhao H, Pei X, Shi H, Sun J, Zhang Y, Wang Z, Zhou M. Metsigdis: a manually curated resource for the metabolic signatures of diseases. Brief Bioinforma. 2017;:bbx103. 10.1093/bib/bbx103 28968812

[38] Peng J, Wang H, Lu J, Hui W, Wang Y, Shang X (2017). Identifying term relations cross different gene ontology categories. BMC Bioinforma.

[39] Peng J, Xue H, Shao Y, Shang X, Wang Y, Chen J (2017). A novel method to measure the semantic similarity of hpo terms. Int J Data Min Bioinforma.

[40] Peng J, Zhang X, Hui W, Lu J, Li Q, Shang X. Improving the measurement of semantic similarity by combining gene ontology and co-functional network: a random walk based approach. BMC Syst Biol. 2018;:12(Suppl2). In press. 10.1186/s12918-018-0539-0 PMC5861498 29560823

[41] Network CGAR (2011). Integrated genomic analyses of ovarian carcinoma. Nature.

[42] Network CGAR (2013). Integrated genomic characterization of endometrial carcinoma. Nature.

[43] Min J, Hemani G, Smith GD, Relton CL, Suderman M. Meffil: efficient normalisation and analysis of very large dna methylation samples. bioRxiv. 2017:125963. 10.1093/bioinformatics/bty476 PMC6247925 29931280

[44] Pidsley R, Zotenko E, Peters TJ, Lawrence MG, Risbridger GP, Molloy P, Van Djik S, Muhlhausler B, Stirzaker C, Clark SJ (2016). Critical evaluation of the illumina methylationepic beadchip microarray for whole-genome dna methylation profiling. Genome Biol.

